# Genetic research with Indigenous Peoples: perspectives on governance and oversight in the US

**DOI:** 10.3389/frma.2023.1286948

**Published:** 2023-11-22

**Authors:** Nanibaa' A. Garrison, Stephanie Russo Carroll

**Affiliations:** ^1^Traditional, ancestral and unceded territory of the Gabrielino/Tongva peoples, Institute for Society and Genetics, University of California, Los Angeles, Los Angeles, CA, United States; ^2^Traditional, ancestral and unceded territory of the Gabrielino/Tongva peoples, Institute for Precision Health, David Geffen School of Medicine, University of California, Los Angeles, Los Angeles, CA, United States; ^3^Traditional, ancestral and unceded territory of the Gabrielino/Tongva peoples, Division of General Internal Medicine and Health Services Research, David Geffen School of Medicine, University of California, Los Angeles, Los Angeles, CA, United States; ^4^Lands of the O'odham and Yaqui peoples, Mel and Enid Zuckerman College of Public Health, University of Arizona, Tucson, AZ, United States; ^5^Lands of the O'odham and Yaqui peoples, Native Nations Institute, Udall Center for Studies in Public Policy, University of Arizona, Tucson, AZ, United States

**Keywords:** Indigenous, data governance, tribal sovereignty, genomic research, ethical research

## Abstract

**Introduction:**

Indigenous Peoples are increasingly exerting governance and oversight over genomic research with citizens of their nations, raising questions about how best to enforce research regulation between American Indian, Alaska Native, and Native Hawaiian peoples and researchers.

**Methods:**

Using a community-engaged research approach, we conducted 42 semi-structured interviews with Tribal leaders, clinicians, researchers, policy makers, and Tribal research review board members about their perspectives on ethical issues related to genetics research with Indigenous Peoples in the US.

**Results:**

We report findings related to (1) considerations for Indigenous governance, (2) institutional relationships upholding sovereignty, (3) expectations for research approvals, and (4) agreements enacting Indigenous governance. Participants described concerns about different ways of exerting oversight, relationships and agreements between Indigenous Peoples and researchers, and gaps that need to be addressed to strengthen existing governance of genomic data.

**Discussion:**

The results will ultimately guide policy-making and development of new strategies for Indigenous Peoples to enforce oversight in research to promote ethically and culturally appropriate research.

## 1 Introduction

Genomic research has been expanding to Indigenous communities, even though Indigenous Peoples remain historically underrepresented in such research globally (Popejoy and Fullerton, 2016; Mills and Rahal, 2019). Underrepresentation of Indigenous Peoples grew out of historical distrust of research and policies, challenges to sovereignty, misalignment of values, cultural concerns, and lack of direct tangible benefits to communities who have participated in previous research (Garrison et al., 2019b; Hiratsuka et al., 2020b). Given these experiences, Indigenous Peoples have raised concerns about unrestricted or unauthorized secondary data uses, stemming from extractive research and exploitation of resources (James et al., 2014; Hudson et al., 2020).

Indigenous Peoples' right to self-determination—that is, their right to freely choose and pursue economic, social, and cultural development goals—was recognized internationally in the 2007 United Nations Declaration on the Rights of Indigenous Peoples (UNDRIP) (United Nations General Assembly, 2007). UNDRIP affirms Indigenous rights to be acknowledged and pursued in the spirit of partnership and respect. In the United States (US), Tribal Nations have developed mechanisms for data governance over genomic resources for their peoples, including research review. In this paper, we use “Indigenous Peoples” as a term to refer to American Indian, Alaska Native, and Native Hawaiian peoples that affirms their political and rights-based statuses. We also use “Tribal Nation” to refer to federally and state recognized Tribes in the continental US and Alaska. Tribes in the US have been exercising control over data and resources from their territories and citizens through laws and policies set by their sovereign governments. At the same time, the rise of Indigenous Data Sovereignty, or the rights of Indigenous Peoples to govern data from and about them, has strengthened oversight over their data and knowledge.

To exert control and oversight over genomic resources, some Indigenous Peoples in the US have banned or have imposed restrictions on certain types of research. For example, concerns about lack of tangible benefits from genetic research as well as a recognition that there is a lack of policies explicitly governing genetic research prompted Navajo Nation to impose a moratorium in 2002 (Navajo Nation Council, 2002). Additionally, a proposal to map a Native Hawaiian genome was met with protests, leading to a moratorium on genetic research by Native Hawaiian people with resolutions to support genetic education (Association of Hawaiian Civic Clubs, 2004; Tauali'i et al., 2014; Arvin, 2015). The Havasupai Tribe's banishment order came in response to egregious research harms over misuse of blood samples, leading to their lawsuit against Arizona Board of Regents (Hart and Sobraske, 2003; Havasupai Tribe of the Havasupai Reservation v. Arizona Board of Regents and Therese Ann Markow, 2009; Garrison, 2012). Other Indigenous Peoples have generally been hesitant to participate in genetics research, but have done so if appropriate protections are in place (Garrison et al., 2019a).

Efforts to respectfully enable genetic research with Indigenous Peoples are underway through the development of stronger governance and oversight mechanisms by Tribes, institutions, and the federal government (Santos, 2008; Hiratsuka et al., 2017; Claw et al., 2018; Carroll D. M. et al., 2021). Tribes as sovereign entities enact laws and rules, and many have review processes that govern research with their people, communities, and lands (Around Him et al., 2019; Carroll et al., 2022a,b; Garba et al., 2023). To strengthen research sovereignty, some Tribes have developed robust memorandums of understanding with institutions and federal agencies that guide relationships, define parameters and protocols, and seek to repair past harms by ensuring future benefits of research for Indigenous Peoples (National Institutes of Health, 2019). Some tribes have their own research review boards while others rely on the Indian Health Service (IHS) institutional review board (IRB) to dictate how human subjects research can take place on Tribal lands, with Tribal citizens, in Tribal communities, or with cultures and knowledges (Around Him et al., 2019; Carroll et al., 2022a). Tribal IRB structures provide oversight, set forth Tribal expectations and requirements, and often require more from researchers than university IRBs, including evidence of community engagement, clauses that outline ownership status of samples and data, and reporting back to communities and oversight boards on a regular basis (Carroll et al., 2022a).

In 2019, the Global Indigenous Data Alliance released the CARE Principles for Indigenous Data Governance (Collective Benefit, Authority to Control, Responsibility, Ethics) to complement the FAIR principles for scientific data management (Findable, Accessible, Interoperable, Reusable) (Wilkinson et al., 2016; Carroll et al., 2020, 2022a; Carroll S. R. et al., 2021). The CARE Principles set forth minimum guidance for researchers, institutions, and governments to enhance Indigenous Peoples' governance of their data and increase their access to data about their people, lands, and communities. While the CARE Principles describe considerations when engaging with Indigenous Peoples' data, they are high-level guidance that always point to regional or local, Tribal Nation protocols for the collection, application, and use of Indigenous Peoples' data. For example, the OCAP^®^ Principles of ownership, control, access, and possession provides a framework for respectful collection and use of First Nations data in Canada (https://fnigc.ca/ocap-training/). OCAP^®^ is a registered trademark of the First Nations Information Governance Center (FNIGC). Critically, the CARE Principles underscore that there is not a one-size fits all approach to data relationships, and that data sharing agreements, data governance protocols, and other data policies and practices must reflect the values and protocols from the Indigenous Peoples that relate or link to those data.

Researchers, funders, and federal agencies have advocated for open, unrestricted access to scientific data to increase its use and applicability across a wide range of topics and for all peoples. The 2022 White House Office of Science and Technology Policy memo on “Ensuring Free, Immediate, and Equitable Access to Federally Funded Research,” also known as the “Nelson Memo,” directs federal agencies with research spending to update their open access policies to require that scientific data be openly accessible and publicly available at the time of scholarly publication or as designated by the agency for those data not appearing in peer-review publications (Nelson, 2022). While the Nelson Memo appears to require free, unrestricted access to data, including Indigenous Peoples' data created or collected during federally-funded research, the reality is that these data should be “as open as possible, as closed as necessary” (European Commission, 2016). “Open” refers to efforts to make data accessible for use and scientific discovery while “closed” upholds individuals' rights to privacy and confidentiality (Landi et al., 2020). We extend the protection of privacy from individuals to collectives, such as Indigenous Peoples, to recognize the complex interlinkages between people and communities and the foundational role of collective privacy in Indigenous knowledge systems and ways of being (Kukutai et al., 2023).

The National Institutes of Health (NIH) implemented new requirements for data management plans pertaining to data sharing, management, and protecting participant privacy, while providing guidance on planning and budgeting for its implementation (National Institutes of Health, 2020, 2023). For example, NIH explicitly underscores that Tribal Nations' laws, policies, protocols, and preferences must be adhered to when designing and implementing open data practices, data sharing agreement, and data management plans (National Institutes of Health, 2022). The NIH Genomic Data Sharing policy requires federally-funded investigators to deposit de-identified data into federal databases to promote secondary analyses (National Institutes of Health, 2014). However, the current policy allows a data sharing exception that recognizes some Tribal Nations' laws may not permit broad data sharing. Some Tribal Nations' laws and policies dictate that all data generated from a research study is property of the Tribe and, when a study ends, all data must be returned to the Tribal Nation (Garrison et al., 2019b; Carroll et al., 2022a). A resulting concern about these data sharing policies is that the allowable exceptions are not clearly understood or recognized by all researchers, institutions, or journal editors. For example, some investigators who have collaborated with Indigenous Peoples to carry out research have expressed concerns about journal editors requesting them to submit data to the federal databases, even when the agreement with the Tribe is to not share data.

This study aims to better understand the views of Indigenous Peoples who are engaged in discussions about genetic research and the questions that it raises about appropriate governance and oversight. Here, we examine perspectives and concerns about research review, Indigenous governance, Indigenous research sovereignty, and oversight mechanisms as they relate to genetic research with Indigenous Peoples in the US.

## 2 Methods

### 2.1 Participant recruitment

Discussions about participation in genomic research have raised numerous questions about how such data should be governed. To understand the views and concerns of Indigenous individuals and allies about genetic research in the US, we recruited Tribal leaders, scientists, health researchers, clinicians, Tribal research review board members, directors of health organizations, and policy analysts to participate in semi-structured interviews as part of a larger study (Garrison et al., 2019a).

Participants were invited if they had engaged in public discussions or scholarship about genetic research with Indigenous Peoples. Participants were recruited in person or via a recruitment email through personal connections, at conferences that discussed genetics and Indigenous Peoples, or through snowball sampling referrals. Approximately 200 individuals were approached at conferences and given information about the study, and 59 individuals were sent recruitment email letters. This study was approved by Seattle Children's Hospital and the University of California, Los Angeles IRBs.

### 2.2 Data collection

Participants were asked to share their perspectives and priorities on genetic research with Indigenous Peoples, including research oversight and data governance to improve Tribal Nations' research codes, guidelines, and policies (Table 1). We report on questions about data governance and research oversight.

**Table 1:** Interview questions.

What types of genetic research studies would you or your Tribe view as appropriate? What types would not be appropriate for you or your Tribe? What steps need to be in place to ensure culturally-sensitive research? What guidelines or policies are necessary to ensure appropriate research? What should researchers do with the data from a study after it has finished? What should researchers do with the results from a study after it has finished?

Semi-structured interviews were designed to last 50 min and were conducted after verbal consent was provided in person or via telephone between June 2016 and May 2018. Participants were compensated for their time with the choice of a $50 gift card or a culturally-relevant gift set (travel mug with an Indigenous Pacific Northwest design and a box of Navajo tea). All interviews were audio recorded and transcribed by a HIPAA-compliant transcriptionist. Transcripts were verified for accuracy and de-identified.

Participants completed a brief demographics survey that included questions about education level, age, gender, self-reported Indigenous affiliation(s), occupation, and self-reported knowledge about genetics. Indigenous identifiers were used to track representation but are not reported to maintain anonymity and respect collective rights. The participants' occupations were reclassified as Tribal leaders, health professionals, or policy experts by the research team to enhance anonymity. Tribal leaders included elected Tribal Nation officials and Tribal elders. Health professionals included scientists, clinicians, nurses, epidemiologists, and public health care workers. Policy experts included policy analysts and Tribal IRB members.

### 2.3 Data analysis

Data collection, analysis, and reporting of findings followed the Standards for Reporting Qualitative Research guidelines (O'Brien et al., 2014). Two coders independently used directed content analysis (Hsieh and Shannon, 2005) to develop a codebook based on the interview guide and coded all interviews with NVivo v.10 software (NVivo, 2014). Coding discrepancies were resolved through discussion or with help from a third coder when needed. We used thematic network analysis to identify and organize emerging themes (Attride-Stirling, 2001).

## 3 Results

### 3.1 Participants

We conducted semi-structured interviews with 42 participants from across the US. Most interviews lasted 50 min but ranged from 30 to 195 min. Thirty-seven (88%) participants reported an affiliation with one or more Tribal Nations and were located across the US, clustered in the Southwest and Pacific Northwest, but did not necessarily live in or near their Tribal Nation. The remaining five (12%) described strong personal and/or professional ties to a Tribal Nation where they had lived or worked for 10 or more years. Demographic information for participants are described in Table 2.

**Table 2:** Demographics.

	***n* (%)**
**Age**	
31–45	14 (33)
46–60	19 (45)
61 and over	9 (21)
**Gender**	
Male	16 (37)
Female	25 (61)
Two-spirit/LGBTQ	1 (2)
**Education level**	
Some college/bachelor's degree	4 (10)
Master's/doctorate degree	38 (90)
**Knowledge about genetics**	
More/much more than others	23 (55)
As much as others	13 (31)
Less/much less than others	6 (14)

### 3.2 Data collection and analysis

Interviews asked about respondents' beliefs, attitudes, and knowledge about research, genetics, genetic research with Indigenous Peoples, and the use of research data. This paper focuses on perspectives about the need for guidelines, policies, and oversight of research with Indigenous Peoples. Themes that emerged focused on (1) considerations for Indigenous governance, (2) institutional relationships upholding sovereignty, (3) expectations for research approvals, and (4) agreements enacting Indigenous governance.

### 3.3 Governance and oversight insights

Tribal leaders, health professionals, and policy experts in this study describe a need for Indigenous Peoples' own guidelines and policies that govern research with their people, land, and communities. Participants note that some Indigenous Peoples may be less willing to participate in research without strong governance in place. At the same time, Indigenous Peoples were grappling with how to ensure the privacy and protection of their people. In general, the participants who were interviewed were concerned about lack of comprehensive policies to govern Indigenous Peoples' data, the extent to which single IRBs would extend to Tribal Nations, and the need for agreements to be in place for all parties engaged in genetic research. Greater discussion among these key players would provide an opportunity to address challenges that researchers face while also trying to build community trust and ultimately, greater inclusion of marginalized or underrepresented communities in research.

Several major themes emerged from the interviews pertaining to the governance structures and processes that govern human research review with Indigenous Peoples.

#### 3.3.1 Considerations for Indigenous governance

Indigenous Peoples in the US govern research through oversight and review processes that include Tribal Nations' research review entities, Tribal colleges and universities, regional review processes, and utilization of other IRBs, such as the Indian Health Service (Around Him et al., 2019; Carroll et al., 2022a). To do so, Tribal Nations have created codes, protocols, and processes, and update those as research, technology, and priorities evolve. One participant described the strategy for developing Indigenous research oversight,

“I am kind of heading up the effort to develop our Institutional Review Board. […] So we're looking at ‘How will we structure it? Who will be part of our Review Board? And how will we look at any research that is presented to us?”' (ID 25, Tribal Leader)

Whether updating or designing new processes, specific considerations for policies relevant to genomics research in the era of open data and data sharing arise such as how data (including specimens) can be used in the future. For example, one participant described a dynamic consent process and the requirement to return data to the community and not to a central repository,

“I think that any time your information is going to be used in a new study, there should be another consent. You know, there should be consent. You know, it should be an active consent, not a passive consent because you've consented once already. […] So there should always be consent requested for any future research, and if the Tribal community wants the data returned to them, it should be returned to them.” (ID 25, Tribal Leader)

The same participant highlighted the need to define data ownership and sharing within Indigenous Peoples' oversight processes, thereby extending governance and control to ownership of data. For example,

“I think the other guideline that we have to think about is […] how research will be shared, how data will be shared, who owns the data? Again, who owns the data? To me, it's the Tribal community that owns the data.” (ID 25, Tribal Leader)

Policies around data ownership, sharing, and consent not only set Indigenous Peoples' expectations for researchers and institutions, they also serve to protect against misuse. Another participant described governing data sharing as a method to mitigate collective harm, but recognized potential impacts of exerting too much control,

“If […] you're trying to control information from flowing, sometimes I think that can actually hurt a whole population of people, especially from a Tribal perspective. […] I wouldn't want our Tribal leaders trying to control the flow of information. I think it would help if people knew about what was going on.” (ID 37, Tribal Leader)

#### 3.3.2 Institutional relationships upholding sovereignty

Before any research projects can start, participants described a need to be aware of existing guidelines and laws for conducting research with Indigenous Peoples globally. These are important for setting the research agenda and relevant laws outline what may be deemed appropriate or not with Indigenous Peoples. For example, one participant described that,

“I think the UNDRIP, the United Nations Declaration of Rights of Indigenous People has some great guidelines as far as, you know, free and informed consent that should be applied. […] Communities just need to know how they can invoke them and use them and not participate, if it's not done in that way that is respectful of what the broad community as a whole wants and needs to have occur in order for them to ensure safety, and I think the number one thing that should be on there is no public dissemination or sharing of any results without consent.” (ID 20, Health Professional)

UNDRIP pertains to Indigenous Peoples within and beyond the US and offers an important framing for research across borders. Within the US, other participants describe a need for state and federal governments to honor and respect Indigenous sovereignty and the existing laws that Tribal Nations have in place. One participant explained,

“Now the state guidelines and the federal guidelines have to honor the Tribal guidelines. […] If you're gonna work in the federally-recognized Native Nation, […] they have to take precedence on what that research means, and the university or institution and federal government or the state have to respect that and they have to work together and come on something that's harmonizing and acceptable both ways, and that's what makes good research is that it's acceptable both ways and the communication is there.” (ID 06, Health Professional)

Another participant elaborated on how it is the responsibility of institutions and researchers to additionally have formal agreements in place. This participant raises concerns about potential future uses of samples in the context of developing agreements, but emphasized that certain agreements need to be in place between the institution and the Tribal Nation. This participant described,

“Researchers come and go, but institutions tend to, by and large, stay around for a long, long time. So if we're talking about controlling future use, […] that means the institution has to control it. […] If the researcher isn't there, isn't alive, you name it, and to do that, [the institution has] to agree to it and they have to sign it. So it's between the institutions, the Tribe or Tribal institution or institutions, and in this case, academic. And the researcher has to agree to it.” (ID 22, Policy Expert)

Finally, another participant described how some universities have formal processes in place for engaging Indigenous Peoples in discussion about research. This participant elaborates on a university-Tribal Nation consultation policy by describing,

“[Our university has] a Tribal consultation policy that any kind of business we do with Tribes that first and foremost, we acknowledge the sovereign status of Tribes, that they're their own separate governments. They have their own laws. They have their own protocols, and basically we need to acknowledge that and we need to honor it and we need to respect it.” (ID 28, Policy Expert)

These tensions and different approaches to implementing policies at the global, national, Tribal, and local levels can pose challenges for researchers in navigating the existing laws and policies, thus these participants describe a need to be aware in order to honor and follow these laws.

#### 3.3.3 Expectations for research approvals

Indigenous Peoples each have different processes and requirements for navigating research oversight to seek and gain support and approvals. Tribal Nations' requirements may be informed by different cultural norms and values, lived histories of the Tribe, experience with prior research, or proximity to research institutions. Some Indigenous Peoples require extensive community engagement, memorandums of agreement or letters of support from community partners, resolutions or other documentation demonstrating community approvals, or formal consultations with Tribal leaders.

One participant voiced a concern about needing additional considerations for genetic research and emphasized the need for tailored consultations with Indigenous Peoples because each Tribal Nation may respond differently to research depending on their experiences, values, or preferences. For example, this participant described that,

“I do think there needs to be more consultation with Tribal communities whenever genetics research is being considered and whenever there are studies, because what may be sensitive to one Tribal community may not be sensitive to another. […] Sometimes it is going to be difficult, because you're gonna need to really engage with multiple communities who may have very differing opinions.” (ID 25, Tribal Leader)

Another participant amplified the rationale for seeking specific approvals about each study separately and explained that cultural expectations across Indigenous Peoples may vary regarding how researchers interact with Elders and individuals. For example,

“It'd have to be defined by each Tribe, of course, ‘cause they might have a very specific way that they'll want an elder interacted with, versus how they might want [a] member of our general population to be interacted with.” (ID 30, Tribal Leader)

Many Indigenous Peoples have their own research review process and requirements that need to be met before any research is approved by the research review board. Indigenous Peoples' processes vary, and it is important for researchers to become knowledgeable about the requirements of the Indigenous Peoples they plan to engage with. Oftentimes, the research review process requires additional steps and approvals by different entities of a Tribal Nations' government or the community that go beyond what most university or external IRBs require. One participant described a process that some Tribal Nations have in place for seeking approval,

“Other Tribes have a policy where your first entry point is at health administration, or the health committee that is staffed by health administration, and you must first get a resolution passed at that level or at the chapter level before it rolls up to the Tribal Council and then has to receive, you know, passage there too.” (ID 21, Health Professional)

In order for Indigenous Peoples to engage in research, a common view was that they need to have codes or frameworks in place that offer protections for their people and cultural knowledges, but some external IRBs may not be familiar with the additional requirements that Indigenous Peoples may have. One participant elaborated by stating,

“Tribes need to have a research code in place that sets a regulatory framework around research that takes place within their jurisdiction. […] So you would have your codes, research process, own local IRB or community review board or some kind of a body that helps oversee any research. […] The codes would already set in place their policies around ownership, around protection of sovereignty, [and] cultural protections. […] So there'd be a whole string of IRB oversight, but those external IRBs do not really concern themselves with local concerns and local priorities, so it would be up to the Tribe to have to do that.” (ID 41, Policy Expert)

Another person explained that Indigenous Peoples need to show support or approval of a project before it even goes to other IRBs, such as the IHS, for additional approvals. Some universities may not approve protocols until the Tribal Nation has or vice versa. For example,

“Our [university] IRB will not approve it until the Tribe has. Same for IHS. Again, it's that respect for the sovereign nation is put in place.” (ID 23, Health Professional)

Thus, the need to understand and respect Indigenous sovereignty was reiterated by several participants who believed that research should not be allowed to proceed without proper steps and approvals in place.

#### 3.3.4 Agreements enacting Indigenous governance

It is common for Tribal Nations to develop and use research agreements that specify requirements and expectations for researchers before engaging in research with Indigenous Peoples. Some of these agreements follow existing policies and are legally binding and others may be developed in the course of setting up research partnerships. In one Tribal Nation, a participant describes that,

“We have a research policy, and then we have a research agreement that we require researchers to sign and it really helps clarify things like who owns the data, what reviews and approvals are required.” (ID 08, Health Professional)

Another participant described a similar process for their Tribal Nation and describes how such an agreement is legally binding and clarifies areas of research that investigators need to follow, including the Tribal Nation's right to review all publications in advance. For example,

“There's a contract piece that's in place in regards to our researcher agreement. So it's a legally binding contract between the principal investigator and our institution that speaks to data owning, biological specimen ownership, management—just agreements that the researcher would have [to agree to]—[to] cede pre-publication review to our Tribal processes.” (ID 24, Health Professional)

Some Tribal Nations specify how data can(not) be used for future studies in their research contracts to clarify upfront what the range of options might be for researchers and define processes for approvals for secondary uses. A participant described,

“We put in our research contracts that they can't use that secondary data without coming back to us first, if they keep it, but we do prefer that it would be either returned or destroyed at the end of the study.” (ID 04, Policy Expert)

This person goes on to elaborate about the need for agreements to be in place before research begins and to allow Indigenous Peoples to review all publications and manuscripts before they are shared widely. This underscores a practice for implementing Indigenous Peoples' control and ownership. Some Tribal Nations have research codes and laws stating that the Indigenous Peoples from whom research data is derived owns that data. One respondent stated,

“There has to be a data-sharing agreement involved in the very beginning of the research so that people know that the Tribe owns the research and Tribe owns the data, […] there has to be understanding in place also that the Tribe has full editorial review of manuscripts and publications and presentations before they leave, before they are seen or disseminated outside of the Tribe.” (ID 04, Policy Expert)

Pre-publication review is not uncommon for Tribal Nations to require in order to review how results are described and contextualized before having manuscripts sent to journals for peer-review or before conference presentations are made. Another participant described practical examples of such review by stating,

“So if, for example, one has as a contractual obligation to a Tribal entity, an understanding that anything to be disseminated, whether it'd be a talk, a poster, a paper, has to go through local Tribal review and approval. From my point of view, that's the essence of control that ownership implies.” (ID 15, Health Professional)

Agreements between Tribal Nations and researchers may go beyond pre-publication review to outline expectations for ownership, control of data, and governance of the overall project. Some agreements specify who is responsible for different aspects of research, as described by one participant,

“Though it says data sharing and ownership agreement, we also talk about co-ownership. […] So the Tribe is a co-owner with the institution. Same thing with control. The Tribe and the institution each are co-controllers, which means that either one can stop a research project.” (ID 22, Policy Expert)

Some participants described protections that should be in place and to allow people the opportunity to decide if they want to be included or not. Some more aspirational goals were to create protections around issues to be addressed for the future, as described by one respondent,

“[It would involve] protections of specimens, protection of data, access, secondary use. [It would have to include] registries and repositories, options, for people to opt in/opt out.” (ID 05, Policy Expert)

Other participants described how research includes different types of knowledge, including cultural or sacred knowledge that needs to be protected. If such data is collected in the course of research, appropriate custodians must be in place to ensure that it remains protected. A respondent described,

“In terms of the data that's collected, who owns it, how it's used, and not just that, but also the cultural knowledge and other types of property. […] It's like a really serious and kind of sacred responsibility to hold that knowledge and to learn as much as you can and to be a custodian of that knowledge, because then you're going to be probably asked to pass it along to other people throughout your lifetime.” (ID 10, Health Professional)

To ensure appropriate Indigenous data sovereignty and governance, some participants described how Tribal Nations need more qualified experts trained in this area, as indicated by a Tribal leader,

“Well, I certainly think there has to be more Native Americans in the medical field, and that includes doctors and nurses and liaisons to be able to communicate this new strategy of being able to work in Native communities.” (ID 37, Tribal Leader)

In summary, respondents shared a range of views and suggestions for improved Indigenous governance and genomic research oversight to facilitate research that may be of benefit to Indigenous Peoples. Respondents noted that Indigenous Peoples may remain hesitant about participation in research if careful attention is not paid to protecting rights and ensuring responsibilities are met.

## 4 Discussion

Meaningful engagement with Indigenous Peoples requires careful attention and adherence to Tribal Nations' laws and research policies. In this study, Tribal leaders, health professionals, and policy experts cited past harms and resulting mistrust of researchers in the context of describing what policies and guidelines are needed to govern genomics research moving forward.

Some participants in this study noted gaps in Tribal Nations' and federal government policies pertaining to how genomic data are governed, stored, and used in secondary analyses. This is consistent with other work indicating gaps in policy coverage that have been identified in an analysis of research legislation, policy, and administrative materials from 26 US Tribal Nations, primarily indicating that expectations for approving research that supports benefit sharing for individuals and the collective, and with a focus on returning research findings, acknowledging Indigenous contributions, supporting a range of economic benefits, and promoting health (Carroll et al., 2022b). Because each Tribal Nation has their own requirements and process for approving and overseeing research, there was no consistent approach for all Tribes. Recommendations by Indigenous and allied scholars have been made to strengthen the protection of Tribal citizens through Tribal law and policy, for researchers to engage in research review processes defined by Tribal governments, and for research institutions to uphold Indigenous rights and ethical principles (Carroll et al., 2022a).

Multiple studies have been conducted to elicit perspectives of Indigenous Peoples as collectives and as individuals about participating in genetics and genomics research. Several key factors relating to appropriate research oversight have been described in the literature as important ways to increase Indigenous Peoples' interest and engagement as collectives and individuals. First, research must be trustworthy and offer reciprocal benefit to participants and their communities (James et al., 2014; Aramoana and Koea, 2019; Brown et al., 2023). Second, relevant policies should align with and respect Indigenous sovereignty, which may require changes to institutions' policies and practices (Claw et al., 2018; Garrison et al., 2019b; Garba et al., 2023). Alignment of policies, budgetary goals, and expectations can happen through the engagement of Indigenous leaders, communities, and scholars (Hoeft et al., 2013). Finally, when research goals and interests are not initially aligned, there is an opportunity for the community to shape and advise the research agenda. For example, a group of pharmacogenomics researchers initially moved toward research on epigenetics and exploration of various social determinants rather than their initial proposed research on pharmacogenomics out of respect for community interests and priorities (Boyer et al., 2011; Morales et al., 2016).

Other Tribal Nations have been actively engaged in genetics and genomics research. For example, the Northwest-Alaska Pharmacogenomics Research Network was established in 2009 and has developed partnerships with Tribal organizations in Montana and Alaska to pursue pharmacogenomic research on pharmacogenes relating to dosing of several common drugs, including warfarin, an anticoagulant drug (Pharmacogenomics Research Network, 2023). The collaboration includes regular partnership meetings for discussions about the partnership, creating best research practices with the community, and identifying challenges as well as developing strategies to improve collaborative efforts with Tribes (Boyer et al., 2011; Shaw et al., 2013). The Bio-Repository for American Indian Capacity, Education, Law, Economics and Technology (BRAICELET) was established in 2015 and partnered closely with the Lakota community, Missouri Breaks Industries Research Inc., Black Hills Center for American Health, and the Stanford Precision Health for Ethnic & Racial Equity Center (SPHERE) to support bi-directional learning and exchange of culture, policy and precision health practices (BRAICELET, 2023; SPHERE, 2023). These efforts built the basis for and eventually transformed into the Native BioData Consortium (NBDC), the first Indigenous-led research institute achieving 501(c)(3) non-profit status in the US. The NBDC biorepository is positioned to conduct and support genetic and health research for the benefit of all Indigenous people (NBDC, 2023).

Discussions about potential benefits of genomic research among Indigenous Peoples, leaders, and researchers often gravitate to addressing concerns, suggesting practical solutions, and highlighting infrastructure needs. The Center for the Ethics of Indigenous Genomic Research (CEIGR) has conducted several deliberations with citizens of Tribal Nations in the US about biobanking, precision health, and genetics research. In a deliberation with Alaska Native community members, participants described having empowerment to make individual choices, improved understanding about factors influencing health, and knowledge as benefits (Hiratsuka et al., 2020a). Conversely, risks included breaches to privacy and confidentiality, discrimination, and even emotional impacts of receiving worrisome results. At Chickasaw Nation, citizens identified potential risks and benefits of participating in genomic research and biobanks, with a particular focus on how data will be controlled and shared to prevent misuses (Reedy et al., 2020). Additionally, the Navajo Nation has had a moratorium on genetics research since 2002. Recent efforts to develop policy has prompted community-engaged work to elicit public perspectives about genetic research and found that the majority of surveyed respondents expressed a need for transparency and cultural considerations about genetics research (Claw et al., 2021).

Beginning in 2017, as study recruitment was happening, multiple discussions were ongoing about large-scale biobanking and data sharing, and the *All of Us* (*AoU*) research program was launched with the intention to enroll Native Americans to address the lack of representation of Indigenous Peoples in large studies (Tribal Collaboration Working Group, 2018; All of Us, 2021). Many participants cited the *AoU* program that raised questions for them about the relevance and appropriateness of over-recruiting Indigenous individuals in efforts to address inequity problems (All of Us, 2021). Because there was an emphasis on Indigenous participants early on, Tribal leaders, health professionals, and policy makers across the US took notice and engaged in discussions or pushed back with concerns about recruitment, control of data, and secondary uses of Indigenous data in a national repository. An *AoU* Tribal Collaboration Working Group wrote a report about conducting consultations with Tribes (All of Us, 2021). Debates ensued about whether the *AoU* program would offer benefit to Indigenous Peoples and critiques about failure to engage Tribal Nations emerged over time (Hansen and Keeler, 2018; Fox, 2020). Thus, some of the participants in this study may have been more concerned about genetic research and framed some of their perspectives about research governance strategies, issues relating to inclusion of Indigenous peoples, and engagement strategies in light of the early *AoU* efforts to recruit Indigenous Peoples in the US.

Some Indigenous Peoples have research review processes in place that add a layer of requirements for researchers to comply with compared to mainstream. For example, the Navajo Nation Human Research Review Board requires the establishment of community partners with documented support of the project as well as return of all data results to Tribe (Brugge and Missaghian, 2006). Other Tribes have requirements in place for ensuring that any royalties from research are given back to the Tribe and protections are in place for intellectual property (Carroll et al., 2022b). Pre-publication review affirms the rights of Indigenous Peoples to ensure that protected or potentially stigmatizing information is not shared with the public through publications or presentations (Hudson et al., 2020; Carroll et al., 2022b). Reasons for additional requirements stem from a number of research missteps, for example, misrepresentation of communities in research and unauthorized, secondary uses of data (McInnes, 2011; Garrison, 2012; Chennells and Steenkamp, 2018; Guedes and Guimaraes, 2020). Failure to appropriately ensure Indigenous Peoples have a role in shaping and reviewing research will lead to missed opportunities to meaningfully engage them in genomics, thus continuing to widen health disparities (West et al., 2017).

Creation, knowledge, and implementation of governance mechanisms that uphold Indigenous sovereignty in research require interventions in both Tribal Nations and other institutions. Research institutions must implement laws, policies, ethics, and infrastructure that uphold Indigenous Peoples own governance, laws, policies, protocols, and preferences. For example, in Australia, the national code of ethics for research with Indigenous Peoples reflects international standards such as the CARE Principles and underscores Indigenous community rights to control research (AIATSIS, 2020). In order to do this, Indigenous Peoples need to adopt relevant and up to date governance that address concerns, such as those expressed by participants in the research presented here, and make governance materials available and accessible via web sites, repositories, and upon request in order for other institutions and individuals to adhere to the requirements (Garba et al., 2023).

## 5 Limitations

A study limitation is that many Tribal leaders declined to participate because they did not feel qualified to actively engage in discussions about genetics and data sharing, so they referred us to other health professionals, many of whom had advanced degrees or experiences with genetic research that may not be generalizable to all American Indian, Alaska Native, and Native Hawaiian leadership. We did not actively recruit community members because we sought expertise from leaders related to genetic research and federal government and Indigenous Peoples' policies. Most recruitment focused on the western half of the US, where more Indigenous representation exists, and therefore did not capture the full range of diverse views from Tribal Nations in the eastern US. While we recognize that this limits the overall generalizability of the findings, our qualitative analysis reveals a wide range of views and experiences about Indigenous data governance, research oversight, and requirements for partnerships between Indigenous Peoples and researchers.

## 6 Conclusion

This qualitative study offers insights into Indigenous Peoples' existing policies and guidelines for researchers as well as identifies gaps that should be addressed to strengthen Indigenous Peoples' oversight and governance of genomic research data. Because American Indian, Alaska Native, and Native Hawaiian leaders have not (been) systematically engaged in federal policy development, opportunities to strengthen nation-to-nation governance and uphold sovereignty have been missed. Steps need to be taken to improve governance and to build pathways forward that implement Indigenous Peoples own laws, policies, protocols, and preferences within federal and other research institutions laws, policies, ethics, and infrastructure.

## Data availability statement

The original contributions presented in the study are included in the article/supplementary material, further inquiries can be directed to the corresponding author.

## Ethics statement

The studies involving humans were approved by Seattle Children's Hospital and University of California, Los Angeles. The studies were conducted in accordance with the local legislation and institutional requirements. The participants provided their informed consent to participate in this study.

## Author contributions

NAG: Conceptualization, Data curation, Formal analysis, Funding acquisition, Writing – original draft, Writing – review & editing. SRC: Formal analysis, Writing – original draft, Writing – review & editing.
